# Xanthones Production in *Gentiana dinarica* Beck Hairy Root Cultures Grown in Simple Bioreactors

**DOI:** 10.3390/plants10081610

**Published:** 2021-08-05

**Authors:** Branka Vinterhalter, Nevena Banjac, Dragan Vinterhalter, Dijana Krstić-Milošević

**Affiliations:** Department of Plant Physiology, Institute for Biological Research “Siniša Stanković”—National Institute of Republic of Serbia, University of Belgrade, Bulevar Despota Stefana 142, 11000 Belgrade, Serbia; horvat@ibiss.bg.ac.rs (B.V.); mitic.nevena@ibissbg.ac.rs (N.B.); dvinterhalter@yahoo.com (D.V.)

**Keywords:** transformed roots, bubble bioreactor, TIS RITA^®^, norswertianin, sucrose

## Abstract

The hairy root clones of *Gentiana dinarica* cl-B, cl-D, cl-3, and cl-14 were cultivated in parallel in diverse simple bioreactors, including temporary immersion systems RITA^®^ (TIS RITA^®^), bubble column bioreactors (BCB), and Erlenmeyer flasks (EF), and evaluated for biomass production and xanthone content. The obtained results showed that TIS RITA^®^ and BCB containing ½ MS medium with 4% sucrose provided equally good growth conditions in which the majority of the clones displayed the higher percentage of dry matter (DM%), and xanthones norswertianin-1-*O*-primeveroside (nor-1-*O*-prim) and norswertianin production than those cultivated in EF. Thin and well branched hairy root clone cl-B grown in BCB for 7 weeks was superior regarding all growth parameters tested, including growth index (19.97), dry weight (2.88 g), and DM% (25.70%) compared to all other clones. Cl-B cultured in TIS RITA^®^ contained the highest amount of nor-1-*O-*prim (56.82 mg per vessel). In BCB with constant aeration, cl-B accumulated the highest norswertianin content reaching 18.08 mg/vessel. The optimized conditions for cultivation of selected *G. dinarica* hairy root clones in highly aerated TIS RITA^®^ and BCB systems contribute to the development of bioreactor technology designed for the large scale commercial production of xanthones nor-1-*O-*prim and norswertianin.

## 1. Introduction

The demands for plant-derived bioactive compounds for healthy food production and for the pharmaceutical industry are permanently increasing making the large scale production of these compounds economically interesting. Despite the significant advance in development of synthetic chemistry techniques, the plants are still the most important source of new bioactive compounds and drugs. The species comprising the Gentianaceae family are interesting for the pharmaceutical industry as they contain numerous compounds with important phytochemical properties [1]. However, as in the case of other medicinal plants, bioactive compounds in Gentians are present in quantities insufficient for pharmaceutical trials and mass production. In addition, many medicinal plant species, such as Gentians, have become endangered in nature due to uncontrolled harvesting, climate changes, and habitat pollution. Nowadays, the technique most commonly used to overcome these problems is biotechnology based on the use of plant tissue culture methods [2,3]. In vitro techniques enable plant cultivation in controlled environmental conditions offering establishment of facilities suitable for large scale production of plant biomass and diverse bioactive compounds [4,5].

*Gentina dinarica* Beck. is a rare and endangered plant species limited to Dinaric mountains of Balkan peninsula and Apennines mountains in Abruzzo Italy. It is characterized by short stem and intensively blue colored flowers. Like the other gentians, *G. dinarica* is rich in secondary metabolites of interest, such as secoiridoid glucosides and particularly xanthones [6].

The majority of xanthones, except in fungi and lichens, were found only in two families of higher plants—Guttiferae and Gentianaceae [7]. Previous phytochemical investigation of *G. dinarica* revealed the presence of 1,3,7,8-oxygenated xanthones, which are typical for *Gentiana* plant species [8]. Recently, Venditti et al. [9] reported for the first time the presence of loganic acid, 6′-*O*-β-D-glucosyl-gentiopicroside, and ursolic acid in the fruits and seeds of *G. dinarica.* Norswertianin-1-*O*-primeveroside and its aglycone norswertianin were detected as the main xanthones in roots of *G. dinarica*. Bioproduction of xanthones is of particular interest since they exhibited a number of very important bioactivities that could be exploited in biomedicine [10]. Thus, norswertianin was shown to display the wide spectrum of bioactivities with antibacterial, antifungal, antioxidative and hypoglycemic effects [11], as well as potential to act as a chemopreventive agent [12]. Recently, anticancerogenic activity of the crude extracts of *G. dinarica* hairy roots and purified norswertianin was also revealed [13].

The protocols for shoot micropropagation, excised root cultures, and hairy root cultures of *G. dinarica* were developed and the data of secondary metabolites content in their tissues have been reported [6,14,15,16]. Among them, the hairy root cultures showed the highest production of bioactive compounds [15]. Compared to plants grown in nature, in vitro cultured plants and tissues had a higher content of xanthones, especially in root and hairy root tissues grown under low light intensities [6,15,17]. Thus, scale-up of hairy root culture using bioreactors provides an opportunity to enhance bioactive compound production at the commercial level [16]. Hairy root cultures were shown to be suitable for xanthone production in *G. dinarica* with several predominant clones selected for further research. These clones were characterized by a different growth rate and phenotype features. Thus, the roots of the clones cl-3 and cl-B were thin and more branched compared to the clones cl-D and cl-14, which were thick and less branched. [15].

Optimal composition of liquid media for sustainable hairy root growth and satisfactory secondary metabolite production was determined in elicited and non-elicited hairy root cultures of *G. dinarica* [18]. Hairy root cultures had increased growth plasticity and stability so that their clones could be maintained for years without a considerable decrease of growth rate. Accordingly, a high biomass production system for hairy roots of *G. dinarica* in hormone-free liquid media can be pointed out as a promising source for the large scale production of xanthones.

In order to maximise production of bioactive components, the optimization of in vitro culture conditions is necessary for each plant species. Supply of oxygen was shown to be a major challenge for the growth of permanent liquid hairy root cultures [19] and different cultivation concepts have been developed using mid- and large scale bioreactors [20]. Therefore, bioreactor aeration was of special interest in the study that we present here.

Many different bioreactors have been created so far, with new designs and improvements still coming out. However, the basic bioreactor types, including those with liquid and gas phase reactors or hybrid reactors, are still frequently used in many studies [21,22,23,24,25]. Apart from hairy roots, micropropagated plants and cell cultures were also used as sources for the production of secondary metabolites in bioreactors. Accumulation of phenolic acids and flavonoids in microshoot cultures of *Schisandra chinensis* was evaluated in different bioreactors [26]. The highest total amount of phenolic acids was found in the cone-type bioreactor, while the highest content of flavonoids was recorded in the nutrient sprinkle bioreactor [26]. Several studies considered bioreactor types suitable for secondary metabolite production from cell suspensions [27,28] and the stirred tank bioreactor [29,30,31], as well as air-lift type bioreactor, have been frequently used.

Therefore, the right choice of bioreactor is an important step as its concept can improve the growth of hairy root cultures in comparison to classic shaken Erlenmeyer flasks and increase the production of secondary metabolites. On the other hand, since the same explant types displayed different growth potential and the ability to produce secondary metabolites [32,33,34], the specific culture conditions are also necessary to be determined in the proposed bioreactors. Accordingly, some bioreactors were designed for specific purposes [35] and a number of them were adapted for some specific plant species and exploitation purposes [21,22,23,24,25,36].

Further enhancement of biomass and of secondary metabolite production in bioreactors can be achieved by the application of biotic and abiotic elicitors [37]. Hairy root cultures were suitable for the treatments with various types of stress inducing elicitors, such as filter-sterilized fungal culture filtrates [35]. Hinposeanahi et al. [38] reported that hairy roots of wild *Vitis vinifera* treated with acetic acid and methyl jasmonate resulted in the highest and lowest amounts of hairy roots biomass and resveratrol content, respectively. Some approaches indicated the mutual effect of simultaneous, side by side application of different elicitors. Thus, Wang et al. [39] found that combined treatment of *Salvia miltiorrhiza* hairy root cultures with ultraviolet-B (UV-B) radiation and methyl jasmonate exhibited synergistic effects on biologically important tanshinone biosynthesis.

In a review covering factors affecting secondary metabolite production, light intensity was not listed as a factor that affects xanthone production and accumulation [40,41,42].

Since *G. dinarica* is a highly endangered species with considerable potential for bioactive compounds production, further screening for in vitro conditions for enhanced biomass and biocompound production convenient for the use in pharmaceutical industry is desirable. The production system optimization required that multiple *G. dinarica* hairy root lines should be included as the yield of the desired products could vary significantly among them [43]. The cultivation conditions also considered the variation of sucrose content in the growth media and supply of oxygen since they can strongly affect hairy root biomass and secondary metabolite accumulation [44].

The present study is a basic and promising approach that points to the enhanced production of hairy root biomass and desired xanthone using dedicated bioreactors under defined conditions. Increasing production of bioactive xanthones allow for further scaled-up research on hairy root propagation and production of pharmacologically active xanthones in large bioreactors in order to further their application in the pharmaceutical/medicinal industry. Several promising hairy root clones of *G. dinarica* were cultivated in parallel to three simple bioreactors, including temporary immersion systems RITA^®^ (TIS Rita), bubble column bioreactors (BCB) with different levels of aeration, and cotton plugged Erlenmeyer flasks (EF) mounted on orbital shakers. This study was intended to establish which of the investigated hairy root clones and bioreactor designs provided the best ratio of root growth versus production and accumulation of xanthones.

## 2. Results

### 2.1. Hairy Root Growth

**Figure 1:**
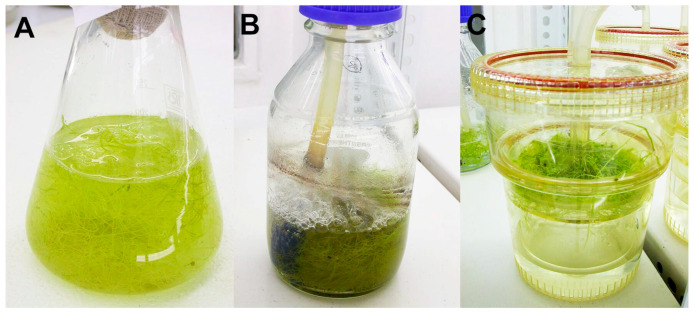
Simple bioreactors: (**A**) Erlenmeyer flask; (**B**) bubble column bioreactor; (**C**) TIS RITA^®^.

To establish the optimal conditions for growth and xanthone production, the hairy roots of *G. dinarica* were cultured in three simple bioreactor systems characterized by diverse aeration mode and media agitation, including Erlenmeyer flasks, bubble column bioreactors and TIS RITA^®^ bioreactors (Figure 1). The hairy root clones cl-B, cl-D, cl-3, and cl-14 displayed different potential for biomass and xanthones production when grown in simple bioreactors containing 200 mL ½ MS medium with 2% or 4% sucrose. According to ANOVA, both growth parameters of hairy root clones, growth index (GI), and dry weight (DW) were significantly affected by the type of hairy root clone, sucrose concentration, bioreactor type, and by their interactions (Table 1).

Dry matter percentage significantly depended on sucrose concentration, bioreactor type, and sucrose concentration x bioreactor type interaction, while the influence of the type of hairy root clone was insignificant.

**Figure 2:**
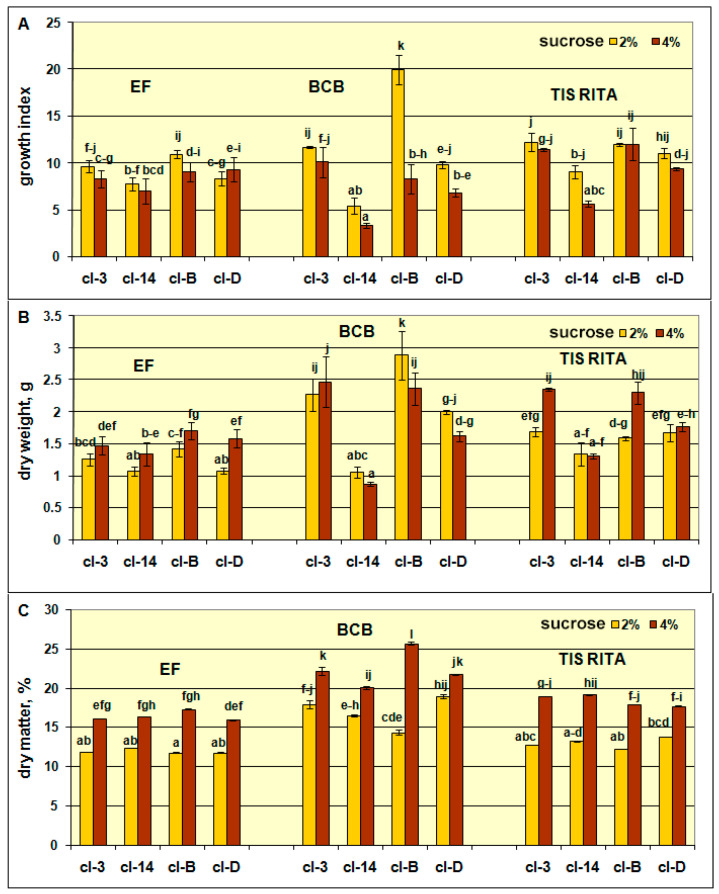
Growth index (**A**), dry weight (**B**), and percentage of dry matter (**C**) of *Gentiana dinarica* hairy root clones cl-3, cl-14, cl-B, and cl-D, after 7 weeks of cultivation in Erlenmeyer flasks (EF), bubble column bioreactor (BCB), and temporary immersion systems (TIS) RITA^®^. Presented values are mean ± SE of 3–6 replicates. Within each parameter, values with the same letter are not significantly different at the *p* ≤ 0.05 level according to Fisher’s least significant difference (LSD) test.

The choice of bioreactor type proved to be significant for the in vitro growth of *G. dinarica* hairy roots. The higher aeration of the explants cultured in TIS RITA^®^ and in BCB with continuous air flow via spargers enabled superior growth as the majority of clones displayed the higher GI, DW, and DM% when compared to EF (Figure 2A–C). Temporary aeration of BCB (20 min per 1 h or 20 min per 4 h) influenced low hairy root growth and finally root tissue necrosis, particularly in the medium with 4% sucrose (Figure S1A,B). Since cl-14 had the lowest growth potential, three out of four tested clones (cl-3, cl-B, cl-D) (Figure 2A–C) were designated as preferable for further cultivation in order to produce secondary metabolites. Additionally, BCB with the medium containing 4% sucrose and with continuous air flow with spargers provided optimal conditions in which preferable clones displayed the highest DM% values (Figure 2C). The use of continuous aeration with spargers resulted in increased media and root agitation, with moderate formation of froth on top of the liquid medium.

The roots of the vigorously growing clones cl-3 and cl-B were thin and more branched than those of lower growing cl-D or the lowest growing cl-14, characterized by thick and less branched roots. The clones showed attributable features required for a long-term cultivation as no changes of the root phenotype after culturing in different treatments was observed. Generally, thin and well branched hairy root clone cl-B grown in BCB could be selected as superior regarding all growth parameters tested, including GI (19.97), DW (2.88 g), and DM% (25.70%) compared to all other clones tested.

### 2.2. The Content of Xanthones Norswertianin-1-O-Primeveroside and Norswertianin

**Figure 3:**
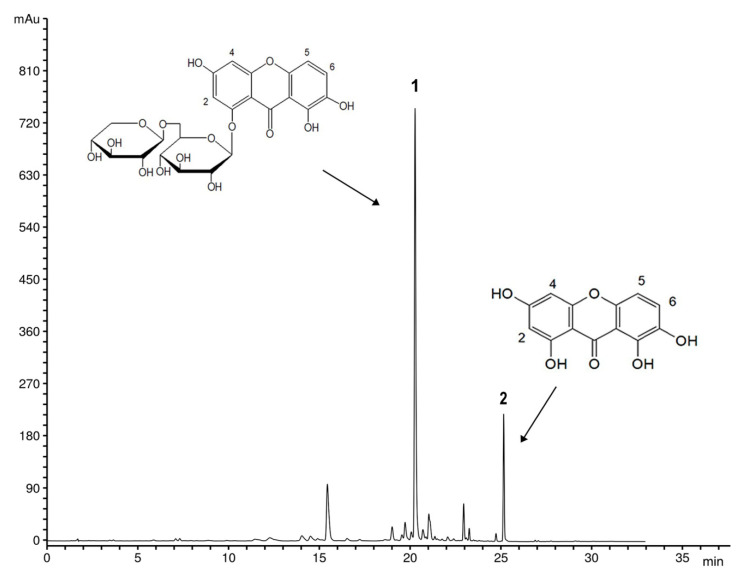
HPLC profile (λ = 260 nm) typical for methanol extract of *G. dinarica* hairy root clones with chemical structures of the main xanthones norswertianin-1-*O*-primeveroside (peak 1) and norswertianin (peak 2).

The influence of bioreactor type and sucrose concentration on the content of nor-1-*O*-prim and norswertianin in four different hairy root clones of *G. dinarica* is presented in Figure 3. The HPLC analysis revealed the presence of both xanthone compounds in all tested clones, with nor-1-*O*-prim as the dominant xanthone.

According to ANOVA, the production of xanthones was affected by the type of *G*. *dinarica* hairy root clones, the sucrose concentration, and the type of bioreactor. Their interaction effects were statistically significant and depended on the type of xanthones studied (Table 2).

**Figure 4:**
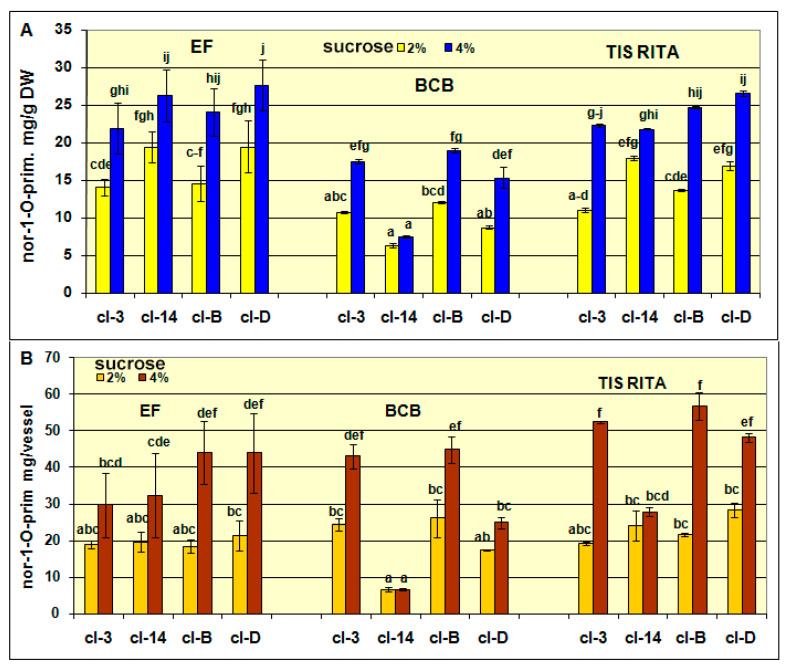
The content of norswertianin-1-*O*-primeveroside in hairy root clones cl-3, cl-14, cl-B, and cl-D, after 7 weeks of cultivation in Erlenmeyer flasks (EF), bubble column bioreactor (BCB) and temporary immersion systems (TIS) RITA^®^. (**A**) The contents are presented as mg/g of dry weight and (**B**) the contents are presented as mg per vessel (bioreactor). Values are expressed as mean ± SE (*n* = 3–5). The different letters above bars denote significant difference by Fisher’s least significant difference (LSD) test, *p* ≤ 0.05.

**Figure 5:**
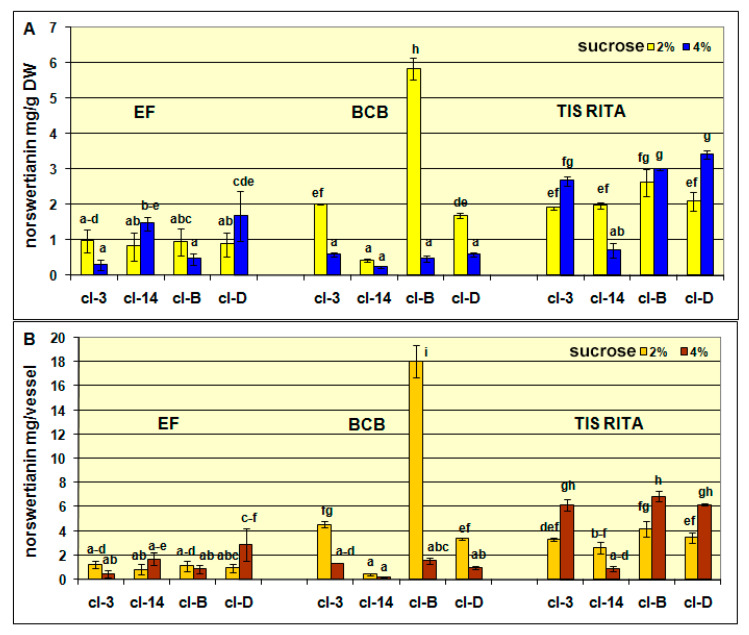
The content of norswertianin in hairy root clones cl-3, cl-14, cl-B, and cl-D, after 7 weeks of cultivation in Erlenmeyer flasks (EF), bubble column bioreactor (BCB) and temporary immersion systems (TIS) RITA^®^. (**A**) The contents are presented as mg/g of dry weight and (**B**) the contents are presented as mg per vessel (bioreactor). Values are expressed as mean ± SE (*n* = 3–5). The different letters above bars denote significant difference by Fisher’s least significant difference (LSD) test, *p* ≤ 0.05.

Hairy roots of cl-14 produced the lowest amount of both xanthones compared to the other clones (Figure 4A and Figure 5A). The clones cl-3, cl-B, cl-D produced more nor-1-*O*-prim in EF and TIS RITA^®^ bioreactors than in BCB (Figure 4A). Nevertheless, due to the high hairy root biomass production in BCB, the differences in the amounts of nor-1-*O*-prim produced per vessel were statistically insignificant regardless of bioreactor used (Figure 4B). It is evident that increasing sucrose concentration from 2% to 4% stimulated nor-1-*O*-prim production for approximately 35–50% in well growing clones (cl-3, cl-B and cl-D), while in slow growing cl-14 this increase ranged from 16–26% depending on bioreactor type. Generally, the highest level of nor-1-*O*-prim was recorded in hairy root clones cultivated at 4% sucrose in bioreactors with higher aeration (BCB and TIS RITA^®^). Thus cl-B grown in TIS RITA^®^ contained the highest amount of nor-1-*O*-prim (56.82 mg per vessel), while the satisfactory yield of this xanthone was also recorded in cl-3 grown in the same type of bioreactor (52.46 mg per vessel).

The clones cl-3, cl-B, and cl-D produced the lowest amount of norswertianin in EF compared to those cultured in higher aerated TIS RITA^®^ and BCB (Figure 5A,B). Unlike nor-1-*O*-prim, the production of aglycone norswertianin was several times higher at 2% sucrose than at 4% in cl-B (12-fold) and cl-3 (3.5-fold) cultured in BCB (Figure 4A,B). The hairy root cl-B previously characterized as a high biomass producing clone has also accumulated the highest norswertianin content, which exceeds 18.08 mg/vessel. The highest production of both xanthones, nor-1-*O*-prim and norswertianin alongside to stable perennial growth, healthy yellow-green color and high biomass production at 2% and 4% sucrose qualify cl-B as the most promising hairy root clone of *G. dinarica.* It is suitable for cultivation in BCB with continuous air flow in order to achieve large scale production of important xanthone compounds. Besides low hairy root biomass production, temporary air flow in BCB bioreactors also influenced low content of both xanthones (Figure S1C–F).

## 3. Discussion

From a practical point of view, the major challenge in the production of plant secondary metabolites is to translate the laboratory production designs into large scale production. The studies of plant biomass and secondary metabolite production in simple bioreactors are the basic and important step toward biochemical production in pharmacological or food industry. Considering that *G. dinarica* is rare and a highly protected plant species with a small area of distribution, cultivation of its hairy roots capable to produce the xanthones at higher levels [15] in bioreactors would be a valid and highly desirable goal.

Hairy roots are an attractive source of phytochemicals due to their proven genetic and biosynthetic stability that offers possibilities for establishing the standards with various parameters, which can affect mass accumulation and bioactive compound production [45]. The selection of valuable hairy root clone/s is also one of the important steps for further optimization of in vitro culture for scaling-up biomass and biochemical production. Accordingly, *G. dinarica* hairy root clones cl-3 and cl-D that have already been appointed as prospects for further research [15] as well as two new clones, cl-B and cl-14, were included in the present study for assessing biomass and metabolite production using three simple bioreactor systems.

By controlling in vitro culture conditions, it is possible to increase the biosynthesis of metabolites of interest [46]. In plant cell and organ cultures, sucrose serves as an energy source for cells and organ proliferation, growth, and metabolite production. Thus, the addition of sucrose to the medium leads to an increase in phenolic and flavonoid compounds in *Vernonia condensata* [47]. The effect of low or high sucrose concentrations on GI and DW parameters on *G. dinarica* hairy roots cannot be generalized since each hairy root clone reacted specifically. However, 4% sucrose provided more compact hairy root cultures and consequently the highest dry matter content. This confirmed benefit of using 4% sucrose for *G. dinarica* hairy roots cultivation as previously stated by Vinterhalter et al. [15]. Alongside, an increase of sucrose concentration (4%) has stimulated production of nor-1-*O*-prim in all bioreactor types as well as norswertianin in EF and TIS RITA^®^. It is indicative that increasing of sucrose concentration, apart from its nutritive effect, generates an osmotic stress triggering a cellular response by enhancing production of defensive secondary metabolites, such as these two xanthones. In adventitious root cultures of *Hypericum perforatum* 3% (*w/v*), sucrose concentration revealed as optimal for root development, since the higher ones have increased osmotic pressure in the medium reducing roots growth [48,49]. However, the highest accumulation of phenolics and flavonoids was recorded in the cultures grown in the medium supplemented with 5% (*w/v*) sucrose.

On the other hand, a significantly higher content of aglycone norswertianin that was recorded in all hairy root clones cultured in BCB at 2% sucrose can partly be explained by the impact of higher aeration rate provided in the BCB compared to other two bioreactors. Some studies reported that a higher aeration rate increased biomass production of adventitious roots, but also decreased secondary metabolite content [50]. However, the majority of studies reported the high aeration rate and constant oxygen supply are important factors enhancing both biomass and metabolite accumulation in the plant cultures grown in bioreactors. By testing different bioreactors used for adventitious root cultivation and saponin production in *Panax ginseng*, Vanĕk et al. [51] found that in well aerated TIS RITA^®^_,_ saponin production was 2.8 times higher compared to roots harvested from less aerated Erlenmeyer flasks. Similarly, significant differences in biomass and secondary metabolite production in hairy root lines of *Centaurium maritimum* cultured in TIS RITA^®^ and EF have been reported by Mišić et al. [52]. Pavlov and Bley [53] also obtained higher biomass production and enhanced betalains content in *Beta vulgaris* hairy roots cultured in a TIS RITA^®^ compared to those grown in EF. Hairy root cultures of *Artemisia annua* grew better in a BCB than in the mist bioreactor and in shaken flasks [21,54] while ginsenosid content in hairy root cultures of *Panax quinquefolium* was two times higher in bioreactors than in shaken Erlenmeyer flasks [55]. Recently, Fortini et al. [44] have also highlighted that the increase in gas exchange rates and sucrose supplementation are efficient strategies for obtaining *Vernonia condensata* plants with both higher biomass and production of flavonoids. *V. condensata* showed higher photosynthetic performance when grown under conditions of greater gas exchange, regardless of sucrose supplementation.

However, Habibi et al. [56] reported that apart from aeration, media, and culture agitation can also be important factors, which enhanced cell growth and increased scopolamine production of *Atropa belladonna* hairy roots in bioreactor. BCB cultivation involves mechanical stress caused by inflows of air and high agitation, which often results in some slight damage of hairy roots that manifests as foaming of growth medium. In *G. dinarica* hairy roots cultured in BCB with spargers, mechanical stress could elicit a defensive cellular response that increased production of norswertianin as a defensive secondary metabolite.

## 4. Materials and Methods

### 4.1. Hairy Root Cultures

Hairy root clones obtained by transforming in vitro grown shoots of *G. dinarica* with *Agrobacterium rhizogenes* strains A4M70GUS (clones cl-B and cl-D) and with 15834/PI (clones cl-3 and cl-14) [15] were used. Hairy root clones were cultured in liquid growth regulator-free MS medium [57] containing ½ inorganic salts (½ MS) and supplemented with 2% (*w/v*) sucrose. pH of the medium was adjusted to 5.8 prior to autoclaving. Long-term hairy root stock cultures were maintained by transferring about 400 mg of fresh weight of the roots into 40 mL liquid medium in 100 mL Erlenmeyer flasks every 5 weeks. Hairy root cultures were then incubated on an orbital shaker (90 rpm min^−1^) in controlled conditions at 25 ± 2 °C and 16 h light/8 h dark photoperiod under dim light (2 μmol m^−2^ s^−1^) provided by cool white fluorescent tubes.

### 4.2. The Hairy Roots Growth in Bioreactors

To eliminate *A. rhizogenes* bacteria after co-cultivation, the hairy roots were subcultured several times on an antibiotic cefotaxim containing media and then finally tested by short culture on a bacterial medium (YEB medium). Free from contamination, hairy root cultures were maintained for several years and therefore there was no influence of bacteria in the production of xanthones.

For all experiments, approximately 1 g fresh weight (FW) of hairy roots (10–25 mm long tips) from 5-week-old stock cultures was inoculated into 200 mL of liquid ½ MS medium containing 2% or 4% (*w/v*) sucrose dispensed in each bioreactor vessel. Three different types of simple bioreactors were used (Table 3): 1—250 mL Erlenmeyer flasks stirred on an orbital shaker; 2—bubble column bioreactor in a 500 mL culture bottle. The medium was aerated with membrane-filtered air up to 0.25 vvm through silicone hose Ø 5 mm or with sparger at the base; 3—temporary immersion systems RITA^®^.

The hairy roots were harvested after 7 weeks of culturing in the bioreactors, washed with distilled water, blotted dry by filter paper towels, and the fresh weight was measured. The roots were dried at room temperature to constant dry weight. Growth index and percentage of dry matter were calculated according to formulas:

GI = (final FW−initial FW)/initial FW(1)

DM% = (DW/FW) × 100(2)

### 4.3. Extraction, HPLC Identification and Quantification of Xanthone Compounds

Xanthone compounds were extracted from dried and powdered hairy roots (500 mg) with methanol (10 mL, J.T. Baker, Deventer, The Netherlands) in ultrasonic bath for 20 min at 30 °C. After sonication, extraction was continued by maceration for 48 h in the dark at room temperature. The extracts were filtered into 10 mL volumetric flasks, adjusted to the volume with methanol, filtered through a 0.45 µm membrane filters, and were used for HPLC analysis. As previously reported by Vinterhalter et al. [15], identification and quantification of xanthones in methanol extracts were performed using Agilent series 1100 HPLC instrument, with a DAD detector, on a reverse phase Zorbax SB-C18 (Agilent) analytical column (150 mm × 4.6 mm i.d., 5 µm particle size) thermostated on 25 °C. The mobile phase consisted of solvent A (1%, *v/v* solution of orthophosphoric acid in water) and solvent B (acetonitrile) using the gradient elution as follows: 98–90% A 0–5 min, 90% A 5–10 min, 90–85% A 10–13 min, 85% A 13–15 min, 85–70% A 15–20 min, 70–40% A 20–24 min, and 40–0% A 24–28 min. The injection volume was 5 µl. Detection wavelengths were set at 260 and 320 nm, and the flow rate was 1 mL min^−1^. Standards of xanthones norswertianin and norswertianin-1-*O*-primeveroside were previously isolated in our laboratory [8,58]. Quantification was performed using a calibration curve with external standards. All experiments were repeated at least two times. The results are presented as milligrams per gram of dry weight.

### 4.4. Statistical Analysis

The results reported are the mean values ± standard error (SE) of 2–4 independent experiments performed in triplicate. The effects of genotype, sucrose concentration and growth condition on the growth parameters change (GI, DW and DM%) and xanthone accumulation in root tissues were evaluated using three-way analysis of variance (ANOVA). Percentage data (DM%) were subjected to angular transformation before statistical analysis, followed by inverse transformation for presentation. Significant differences between means of each treatment were determined by Fisher’s least significant difference (LSD) test at *p* ≤ 0.05 using StatGraphics Plus software package for Windows 2.1 (Statistical Graphics org., Rockville, MD, USA).

## 5. Conclusions

To conclude, this study provides the first report on the influence of bioreactor type on the biomass and xanthones production in *G. dinarica* hairy root cultures. TIS RITA^®^ and BCB bioreactors providing advanced aeration and moderate media agitation, enabled good hairy root growth and secondary metabolite accumulation compared to shaken EF bioreactors, which are inexpensive and require less manual labor for batch preparation and maintenance. The highest amount of nor-1-*O*-prim (56.82 mg per vessel) was recorded in cl-B grown in TIS RITA^®^ at 4% sucrose. When cl-B was cultured in BCB at 2% sucrose, multiple increases occurred in the content of aglycone norswertianin, reaching 18.08 mg/vessel. The obtained results are important for further studies involving scale-up experiments to ensure conditions for commercial production of these xanthones using large scale bioreactors

## Figures and Tables

**Table 1:** Results of nested ANOVA for the effects of clone (A), sucrose concentration (B), and bioreactor type (C) on the growth parameters (growth index, dry weight, and % of dry matter) in hairy root cultures of *Gentiana dinarica.* The bold values indicate statistically significant results (*p* ≤ 0.05).

Source	Sum of Squares	df	Mean Square	*F*-Ratio	*p*-Value
**Growth index**					
(A) clone	397.650	3	132.550	28.702	**0.000000**
(B) sucrose conc.	120.509	1	120.509	26.094	**0.000002**
(C) Bioreactor type	40.192	2	20.096	4.352	**0.015641**
A × B	42.970	3	14.323	3.102	**0.030496**
A × C	162.809	6	27.135	5.876	**0.000032**
B × C	80.908	2	40.454	8.760	**0.000329**
A × B × C	113.653	6	18.942	4.102	**0.001082**
Error	424.871	92	4.618		
Dry weight, g					
(A) clone	11.1355	3	3.7118	37.352	**0.000000**
(B) sucrose conc.	0.4150	1	0.4150	4.176	**0.043854**
(C) Bioreactor type	8.2620	2	4.1310	41.570	**0.000000**
A × B	0.4101	3	0.1367	1.376	0.255190
A × C	6.8783	6	1.1464	11.536	**0.000000**
B × C	2.1979	2	1.0989	11.058	**0.000050**
A × B × C	1.5243	6	0.2540	2.556	**0.024630**
Error	9.1425	92	0.0994		
Dry matter, %					
(A) clone	0.00009	3	0.00003	0.05	0.986045
(B) sucrose conc.	0.11238	1	0.11238	177.86	**0.000000**
(C) Bioreactor type	0.10788	2	0.05394	85.37	**0.000000**
A × B	0.00834	3	0.00278	4.40	**0.006133**
A × C	0.00442	6	0.00074	1.17	0.330999
B × C	0.00029	2	0.00015	0.23	0.792625
A × B × C	0.00816	6	0.00136	2.15	0.054822
Error	0.05813	92	0.00063		

**Table 2:** Results of nested ANOVA for the effects of clone (A), sucrose concentration (B), and bioreactor type (C) on the content of norswertianin-1-*O*-primeveroside and norswertianin in hairy root cultures of *Gentiana dinarica.* The bold values indicate statistically significant results (*p* ≤ 0.05).

Source	Sum of Squares	df	Mean Square	*F*-Ratio	*p*-Value
Nor-1-*O*-prim mg/g DW					
(A) clone	93.15	3	31.05	3.211	**0.030046**
(B) sucrose conc.	998.08	1	998.08	103.206	**0.000000**
(C) Bioreactor type	1270.54	2	635.27	65.690	**0.000000**
A × B	76.01	3	25.34	2.620	0.060073
A × C	451.64	6	75.27	7.784	**0.000005**
B × C	45.93	2	22.97	2.375	0.102691
A × B × C	19.85	6	3.31	0.342	0.911397
Error	52.22	54	9.67		
Nor-1-*O*-prim mg/vessel					
(A) clone	2502.18	3	834.06	9.5775	**0.000036**
(B) sucrose conc.	5380.79	1	5380.79	61.7873	**0.000000**
(C) Bioreactor type	1288.98	2	644.49	7.4007	**0.001444**
A × B	1079.96	3	359.99	4.1337	**0.010380**
A × C	2097.28	6	349.55	4.0138	**0.002146**
B × C	434.09	2	217.05	2.4923	0.092188
A × B × C	465.26	6	77.54	0.8904	0.508505
Error	4702.62	54	87.09		
Norswertianin mg/g DW					
(A) clone	15.9968	3	5.3323	23.3371	**0.000000**
(B) sucrose conc.	5.3186	1	5.3186	23.2774	**0.000012**
(C) Bioreactor type	19.1415	2	9.5708	41.8873	**0.000000**
A × B	11.6281	3	3.8760	16.9639	**0.000000**
A × C	24.9380	6	4.1563	18.1906	**0.000000**
B × C	22.5442	2	11.2721	49.3333	**0.000000**
A × B × C	21.7271	6	3.6212	15.8484	**0.000000**
Error	12.3384	54	0.2285		
Norswertianin mg/vessel					
(A) clone	169.2479	3	56.4160	38.8231	**0.000000**
(B) sucrose conc.	25.9189	1	25.9189	17.8363	**0.000093**
(C) Bioreactor type	122.7504	2	61.3752	42.2359	**0.000000**
A ×B	84.6202	3	28.2067	19.4107	**0.000000**
A × C	232.2130	6	38.7022	26.6332	**0.000000**
B × C	208.5777	2	104.2889	71.7672	**0.000000**
A × B × C	238.6116	6	39.7686	27.3671	**0.000000**
Error	784703	54	1.4532		

**Table 3:** Simple bioreactor types and the growth conditions.

Bioreactor Type	Initial Explant Weight	Medium Volume	Aeration Conditions	Medium Addition	Lighting Conditions
Erlenmeyer flasks	1 g	200 mL	continuous shaking at 90 rpm	No	2 μmol m^−2^ s^−1^ *
Bubble column bioreactor	1 g	200 mL	continuous air blowing through a sparger	Adding sterile deionized water and ½ MS medium every 7 days alternately	40 μmol m^−2^ s^−1^
Bubble column bioreactor	1 g	200 mL	air-blowing 20 min/1 h through hose Ø 5 mm	No	40 μmol m^−2^ s^−1^
Bubble column bioreactor	1 g	200 mL	air-blowing 20 min/4 h through hose Ø 5 mm	No	40 μmol m^−2^ s^−1^
Temporary immersion systems RITA^®^	1 g	200 mL	Immersion 20 min/8 h	No	40 μmol m^−2^ s^−1^

* Preliminary study conducted with clone cl- 3 cultured in shaken EF type bioreactors on white light at 40 μmol m^−2^ s^−1^ showed that light had little effect on growth and biomass production of hairy roots and their accumulation of xanthones.

## Data Availability

The data presented in this study are available from the authors.

## Supplementary Materials

The following are available online at https://www.mdpi.com/article/10.3390/plants10081610/s1, Figure S1: Influence of air blowing frequency (20 min/1 h and 20 min/4 h) on growth index (A), dry weight (B), and content of norswertianin-1-*O*-primeveroside and norswertianin expressed as mg/g of dry weight (C,D), and mg per vessel (E,F), in hairy root clones cl-3, cl-14, cl-B, and cl-D, after 7 weeks of cultivation in bubble column bioreactor. Values are expressed as mean ± SE (*n* = 3–5). The different letters above bars denote significant difference by Fisher’s least significant difference (LSD) test, *p* ≤ 0.05.
