# Supplementary material for: Xanthones Production in Gentiana dinarica Beck Hairy Root Cultures Grown in Simple Bioreactors
Source: Plants (Basel). 2021 Aug 5;10(8):1610. doi: 10.3390/plants10081610 (PMC8401843; doi:10.3390/plants10081610)
Supplement: Supplementary file 1 [file plants-10-01610-s001.zip › plants-1222121-supplementary.pdf]

plants

MDPI

Supplementary file

Xanthones Production in *Gentiana dinarica* Beck Hairy Root Cultures Grown in Simple Bioreactors

Branka Vinterhalter, Nevena Banjac, Dragan Vinterhalter and Dijana Krstić-Milošević *

[1] Department of Plant Physiology, Institute for Biological Research "Siniša Stanković"—National Institute of Republic of Serbia, University of Belgrade, Bulevar despota Stefana 142, 11000 Belgrade, Serbia; horvat@ibiss.bg.ac.rs (B.V.); mitic.nevena@ibissbg.ac.rs (N.B.); dvinterhalter@yahoo.com (D.V.)

* Correspondence: dijana@ibiss.bg.ac.rs; Tel.:+38-11-1207-8394

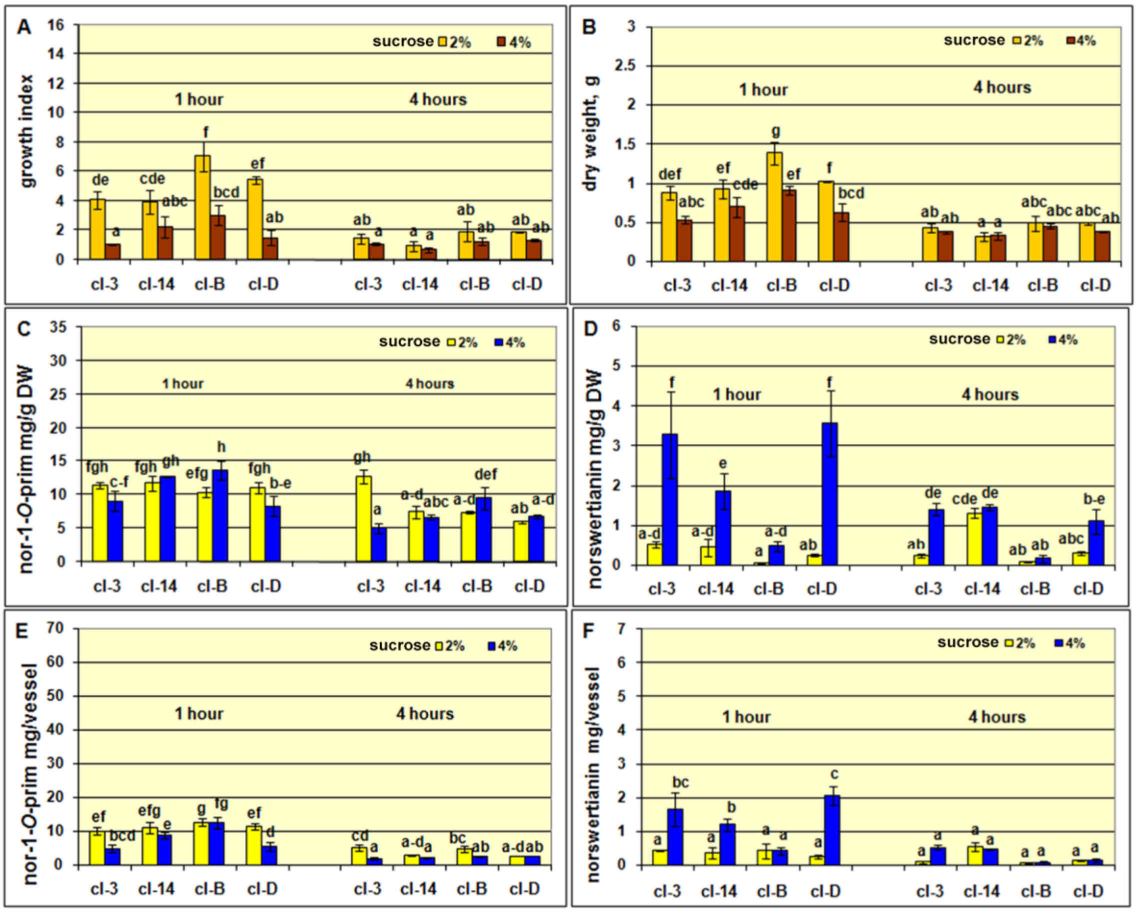

Figure S1. Influence of air blowing frequency (20 min/1 h and 20 min/4 h) on growth index (**A**), dry weight (**B**), and content of norswertianin-1-*O*-primeveroside and norswertianin expressed as mg/g of dry weight (**C**,**D**), and mg per vessel (**E**,**F**), in hairy root clones cl-3, cl-14, cl-B, and cl-D, after 7 weeks of cultivation in bubble column bioreactor. Values are expressed as mean ± SE (n = 3-5). The different letters above bars denote significant difference by Fisher's least significant difference (LSD) test, $p \leq 0.05$.
